# CRISPR/Cas9-mediated targeted mutagenesis of *GmLHY* genes alters plant height and internode length in soybean

**DOI:** 10.1186/s12870-019-2145-8

**Published:** 2019-12-18

**Authors:** Qun Cheng, Lidong Dong, Tong Su, Tingyu Li, Zhuoran Gan, Haiyang Nan, Sijia Lu, Chao Fang, Lingping Kong, Haiyang Li, Zhihong Hou, Kun Kou, Yang Tang, Xiaoya Lin, Xiaohui Zhao, Liyu Chen, Baohui Liu, Fanjiang Kong

**Affiliations:** 1 School of Life Sciences, Guangzhou University, Guangzhou, China; 2 The Innovative Academy of Seed Design, Key Laboratory of Soybean Molecular Design Breeding, Northeast Institute of Geography and Agroecology, Chinese Academy of Sciences, Harbin, China; 3 University of Chinese Academy of Sciences, Beijing, China

**Keywords:** CRISPR/Cas9, Plant height, Soybean, LHY, Transgene-free

## Abstract

**Background:**

Soybean (*Glycine max*) is an economically important oil and protein crop. Plant height is a key trait that significantly impacts the yield of soybean; however, research on the molecular mechanisms associated with soybean plant height is lacking. The CRISPR (clustered regularly interspaced short palindromic repeat)/Cas9 (CRISPR-associated system 9) system is a recently developed technology for gene editing that has been utilized to edit the genomes of crop plants.

**Results:**

Here, we designed four gRNAs to mutate four *LATE ELONGATED HYPOCOTYL* ***(****LHY*) genes in soybean. In order to test whether the gRNAs could perform properly in transgenic soybean plants, we first tested the CRISPR construct in transgenic soybean hairy roots using *Agrobacterium rhizogenes* strain K599. Once confirmed, we performed stable soybean transformation and obtained 19 independent transgenic soybean plants. Subsequently, we obtained one T_1_ transgene-free homozygous quadruple mutant of *GmLHY* by self-crossing. The phenotypes of the T_2_-generation transgene-free quadruple mutant plants were observed, and the results showed that the quadruple mutant of *GmLHY* displayed reduced plant height and shortened internodes. The levels of endogenous gibberellic acid (GA3) in *Gmlhy1a1b2a2b* was lower than in the wild type (WT), and the shortened internode phenotype could be rescued by treatment with exogenous GA3. In addition, the relative expression levels of GA metabolic pathway genes in the quadruple mutant of *GmLHY* were significantly decreased in comparison to the WT. These results suggest that *GmLHY* encodes an MYB transcription factor that affects plant height through mediating the GA pathway in soybean. We also developed genetic markers for identifying mutants for application in breeding studies.

**Conclusions:**

Our results indicate that CRISPR/Cas9-mediated targeted mutagenesis of four *GmLHY* genes reduces soybean plant height and shortens internodes from 20 to 35 days after emergence (DAE). These findings provide insight into the mechanisms underlying plant height regulatory networks in soybean.

## Background

Soybean is one of the most important economic sources of vegetable oil and protein worldwide, and plant height, node number, internode length, branch number, and seed size are significant factors that affect soybean yield [1, 2]. Plant height is a key trait of plant ideotypes, and a relatively shorter stem length contributes to increased yield in modern breeding programs [3–5]. Some plant height genes have thus been cloned by map-based cloning in several plant species, such as maize [6–8], rice [9–11], tomato [12], and soybean [13, 14]. For example, *GA3 b-hydroxylase* (*ZmGA3ox2*) was cloned using candidate gene association mapping and a genetic assay from the dwarf mutant *d1–6016* and responded for the dwarf mutant in maize [7]. The *Brachytic2* (*Br2*) gene, which was cloned from maize by mapping, significantly impacts plant height [8]. Recent research showed that *GmDW1* (dwarf mutant) encodes an ent-kaurene synthase, and the mutant of *GmDW1* displayed reduced plant height and shortened internodes in soybean [13]. In addition, several transcription factor (TF) families play important roles in plant height. For instance, OsNAC2 is a NAC transcription factor, and the constitutive expression of *OsNAC2* resulted in shorter internodes and shorter spikelets in rice [15].

Circadian clocks are endogenous 24-h oscillators that allow organisms to anticipate daily changes in their environment, playing critical roles in many biological processes and stress responses by regulating up to 80% of the transcriptome in plants [16–18]. LHY and CCA1 are key components of the central oscillator and encode two morning-expressed MYB TFs in *Arabidopsis* [19, 20]. AtLHY/CCA1 can bind to the evening element (EE; AAATATCT) of the promoter of *TIMING OF CAB EXPRESSION 1* (*TOC1*) and act redundantly to repress the transcription of the *AtTOC1* gene during the day [21]. AtTOC1 represses *AtCCA1* and *AtLHY* from its induction at dusk until slightly before dawn [22]. Other functions of LHY/CCA1 in flowering and the stress response have been reported [23, 24]. For example, silencing of *NaLHY* abolished the vertical movement of flowers under continuous light conditions in *Nicotiana* [23]. A recent report showed that AtLHY can regulate the expression of abscisic acid (ABA) signaling components and downstream response genes to potentiate some ABA responses [24]. However, the potential functions of the *LHY/CCA1* family members in soybean are still unclear.

The CRISPR/Cas9 system was recently engineered for the genetic manipulation of plants [25–28]. The use of CRISPR/Cas9 technology has attracted great attention and has been successfully applied in various crops for genome editing, such as wheat [29, 30], maize [31, 32], rice [33], barley [34], tomato [35, 36], and soybean [37–39]. There are four *GmLHY* genes in soybean, named *GmLHY1a*, *GmLHY1b*, *GmLHY2a,* and *GmLHY2b*, but the functions of these genes remain unknown. Therefore, in the current study, the CRISPR/Cas9 system was used to target four *GmLHY* genes in soybean. We observed the phenotype of the T_2_-generation transgene-free quadruple mutant of *GmLHY* and found that the height and internodes of the quadruple mutant were significantly shorter than that of the WT. Moreover, the relative expression levels of GA metabolic pathway genes in the quadruple mutant of *GmLHY* were significantly lower than in WT. These results suggested that *GmLHY* directly or indirectly regulates plant height by mediating key components of the GA pathway. We also developed genetic markers for the identification of mutants for use in breeding studies. Our findings suggest that the manipulation of these genes should facilitate improvements in plant height and internodes in soybean.

## Results

### Target site selection, construction, and confirmation of the target sites in soybean hairy roots

**Fig. 1:**
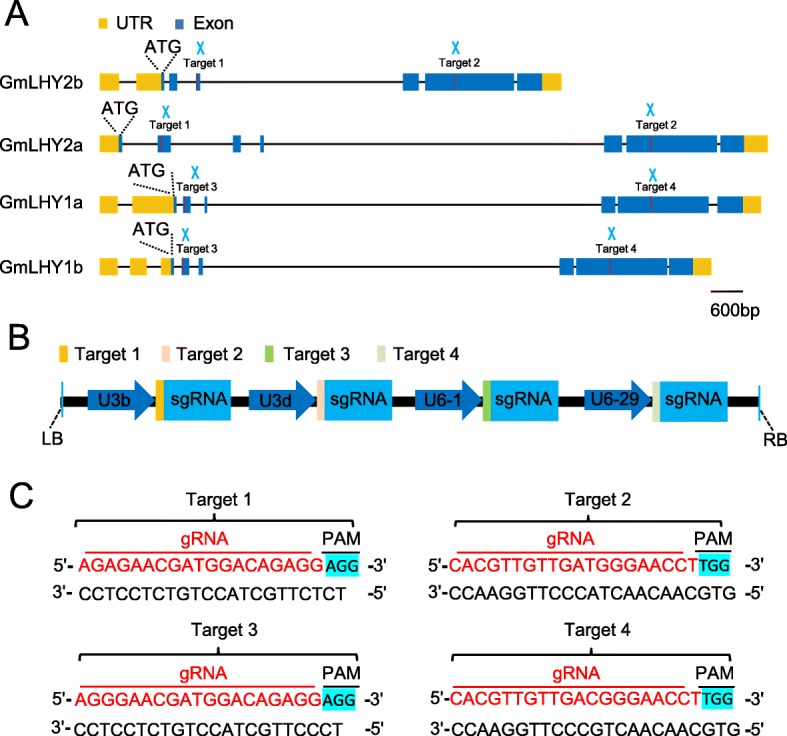
Diagram of the dual gRNA CRISPR/Cas9 vector, target sequences, and target locations of the four *GmLHY* genes. **a** Location of the dual target sites in *GmLHY1a*, *GmLHY1b*, *GmLHY2a* and *GmLHY2b*. Bar = 600 bp; **b** Schematic figure of the binary vector designed for mutagenesis of the *GmLHY* genes using the CRISPR/Cas9 technology; **c** Target sequences

In order to identify the ortholog of AtLHY and AtCCA1 in soybean, we performed protein sequence alignment and identified four CCA1/LHY orthologs in soybean. Phylogenetic analysis showed that the four CCA1/LHY orthologs are closer to AtLHY than AtCCA1. Thus, the four CCA1/LHY orthologs was named GmLHY1a (*Glyma.16G017400*), GmLHY1b (*Glyma.07G048500*), GmLHY2a (*Glyma.19G260900*), and GmLHY2b (*Glyma.03G261800*) (Additional file 1: Fig. S1). To study the function of the four *GmLHY* genes in soybean, four target adaptors were used, including target 1/2 for targeting the *GmLHY2a* and *GmLHY2b* genes, and target 3/4 for targeting the *GmLHY1a* and *GmLHY1b* genes (Fig.1a). Target 1 is present in the second and third exon of the *GmLHY2b* and *GmLHY2a* genes, respectively; target 2 is present in the fifth and sixth exon of the *GmLHY2b* and *GmLHY2a* genes, respectively; target 3 is present in the first exon of *GmLHY1a* and *GmLHY1b*; and target 4 is present in the fifth exon of *GmLHY1a* and *GmLHY1b* in soybean (Fig. 1a). The CRISPR vector encodes Cas9 and was driven by the CaMV35S promoter and four gRNAs driven by the *Arabidopsis* U3b, U3d, U6–1, and U6–29 promoters, respectively (Fig. 1b, c).

In order to test whether the CRISPR/Cas9 construct could properly edit these genes in transgenic soybean plants, we first tested the construct in transgenic soybean hairy roots using *A. rhizogenes* K599 (Additional file 1: Fig. S2A). The transgenic soybean hairy roots were generated by high-efficiency *Agrobacterium rhizogenes*-mediated transformation [40]. When the hairy roots generated at the infection site were approximately 2 cm long, they were used for genotype detection. The genotype of the transgenic hairy roots was detected by PCR using *Cas9* gene-specific primers and *GmLHY* gene-specific primers. We detected mobility-shifted bands in six DNA-bulked samples when the *Cas9* gene-specific primers were utilized. The result showed that there were five transgenic lines with the *Cas9* gene product (*Cas9* gene-positive) (Additional file 1: Fig. S2B). Sequencing analysis of the *GmLHY* genes showed that the *Cas9* gene-positive lines (R1–R5) produced superimposed peaks in the target 1/3 site, while the target 2/4 site was unchanged (Additional file 1: Figure S2C, Additional file 2: Table S1). Together, these results indicated that the transgene-encoded Cas9 and gRNAs were able to efficiently induce double-strand breaks at the target 1/3 sites in the *GmLHY* genes.

### Transgene-free homozygous quadruple mutant of *GmLHY* in soybean

**Fig. 2:**
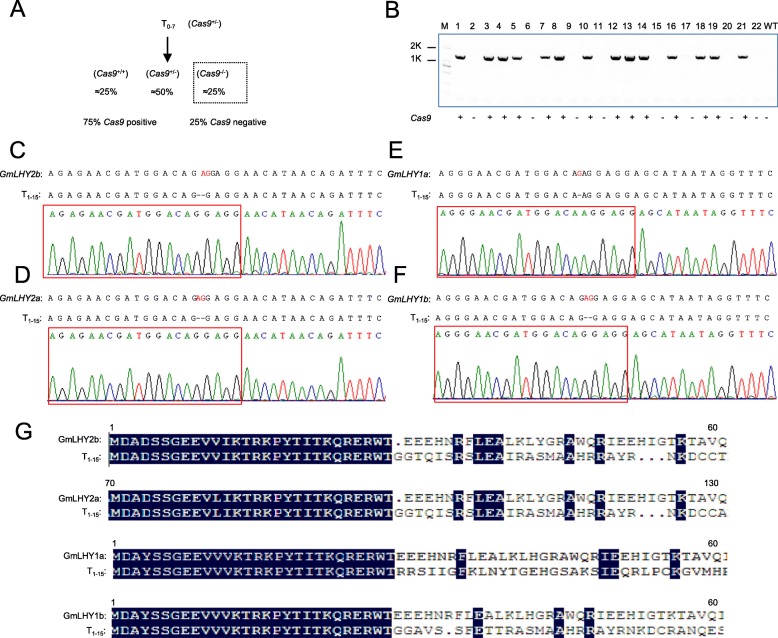
Homozygous targeted mutagenesis of *GmLHY1a/1b/2a/2b* induced by CRISPR/Cas9. **a** Self-crossed scheme for generating a homozygous mutant that contains no Cas9 vector. (+/+) indicates that *Cas9* was homozygous. (+/−) indicates that *Cas9* was heterozygous. (−/−) indicates that *Cas9* was deleted; **b** PCR-based genotyping results for the *Cas9* gene in the T1 generation. (+) indicates that the gene was detected, (−) indicates that the gene was not detected; **c** Detailed sequence of the target site *GmLHY2b* in the T1–15 line; **d** Detailed sequence of the target site *GmLHY2a* in the T1–15 line; **e** Detailed sequence of the target site *GmLHY1a* in the T1–15 line; **f** Detailed sequence of the target site *GmLHY1b* in the T1–15 line. ‘-’ represents the number of deleted nucleotides. The red frames indicate the location of the targets; **g** Multiple alignment of the amino acid sequences of the quadruple mutant of *GmLHY*

We next performed stable soybean transformation and obtained 19 independent T_0_ transgenic lines with the section for the *Cas9* gene product (*Cas9* gene-positive) (Additional file 1: Fig. S3A). Sequencing analysis showed that the T_0_–7 line was a heterozygous quadruple mutant of *GmLHY* that might possess a 2-bp deletion in *GmLHY2b/2a/1b-*target1/3 and a 1-bp deletion in *GmLHY2a-*target3 (Additional file 1: Figure S3B-E; Additional file 3: Table S2). In order to use the mutants in crop breeding, we sought homozygous quadruple mutants of the *GmLHY* line without the transgene and screened the T_1_ plants derived from the T_0_ transgenic lines. Fortunately, we obtained eight T_1_ plants derived from T_0_–7 that lacked the *Cas9* gene (Fig. 2a, b), and only one line (T_1_–15) was a transgene-free homozygous quadruple mutant of *GmLHY* (Fig. 2c–f; Additional file 3: Table S2). Sequencing analysis showed that the quadruple mutant of *GmLHY* had a 2-bp deletion in *GmLHY2b/2a/1b*-target1/3 and a 1-bp deletion in *GmLHY1a*-target3 (Fig. 2c–2f), resulting in frame-shift mutations in the *GmLHY* genes (Fig. 2g).

### The expression level of *GmLHY* in the quadruple mutant and WT

**Fig. 3:**
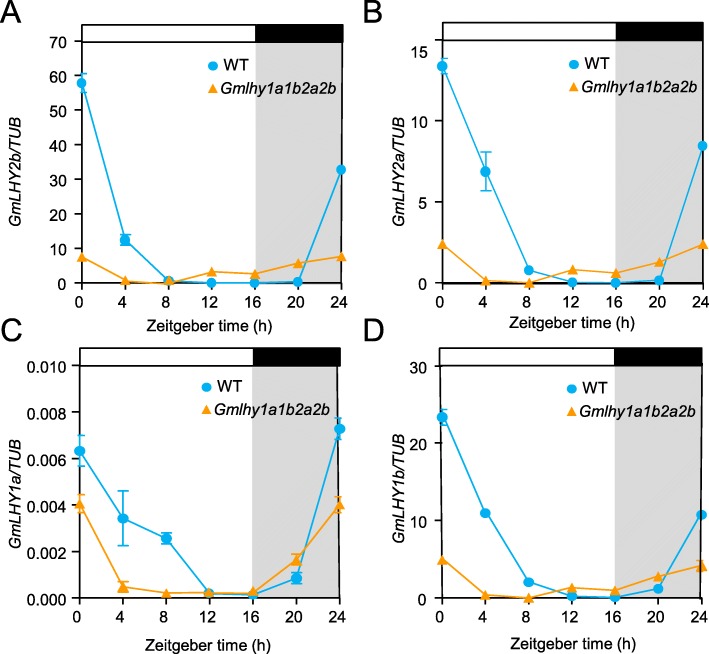
Diurnal expression patterns of *GmLHY1a/1b/2a/2b* in WT plants and T2 homozygous quadruple mutants of *GmLHY*. **a**–**d** qRT-PCR analysis of *GmLHY2b*, *GmLHY2a, GmLHY1a,* and *GmLHY1b* expression levels in the leaves at 20 DAE under 16 h light/8 h dark (long day; LD) conditions, respectively. Data shown are relative to the control gene *GmTUB* and represent means ± standard error of the mean (s.e.m.) for three biological replicates. Bars indicate the s.e.m. Black and white bars represent dark and light periods, respectively

LHY/CCA1 are key components of the circadian clock and participate in the temporal organization of biological activities and the regulation of gene expression [16, 17, 21]. Previous studies have shown that the expression level of *LHY/CCA1* was much higher in the morning than in the night [21]. However, the expression pattern of *GmLHY* genes in the quadruple mutant of *GmLHY* is not known. The diurnal circadian rhythm of *GmLHY* gene expression in the quadruple mutant of *GmLHY* was analyzed by quantitative real-time PCR (qRT-PCR) under inductive long-day (LD) conditions. The result showed that *GmLHY1a*, *GmLHY1b*, *GmLHY2a,* and *GmLHY2b* were highly up-regulated in WT, and the highest expression was detected at 0 h and 24 h after dawn (Fig. 3a–d). However, the expression of *GmLHY* genes was lower in the quadruple mutant of *GmLHY* than WT (Fig. 3A–D). These results showed that the expression of the four *GmLHY* genes was significantly decreased in the quadruple mutant of *GmLHY*.

### The quadruple mutant of *GmLHY* reduces soybean plant height and shortens internodes

**Fig. 4:**
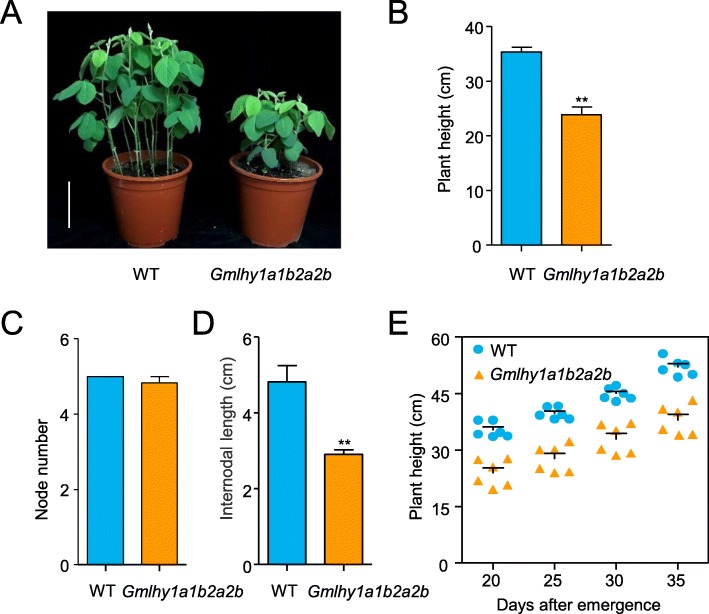
The phenotype of the WT plants and T2 homozygous quadruple mutant of *GmLHY*. **a** The plant height of homozygous T2 seedings and WT; **b** The statistics of plant height for 20 DAE under LD conditions; **c** The statistics of node number for 20 DAE under LD conditions; **d** The statistics of intermodal length for 20 DAE under LD conditions; **e** The statistics of plant height from 20 to 35 DAE. The experiment was performed using six biological replicates, and differences were statistically analyzed using Student’s *t-*test (**P < 0.01). Bars indicate the s.e.m. All data are shown as means ± s.e.m. (*n* = 6 plants)

To examine the loss function of *GmLHY*, the phenotypes of the T_2_-generation transgene-free quadruple mutant and WT plants were observed. We found that the plant height of the quadruple mutant was significantly lower than WT under LD conditions for 20 DAE (Fig. 4a, b). Subsequently, we examined the node number and internodal length, as these impact plant height [13, 15]. As indicated in Fig. 4c and d, the node number did not change, while the internodal length was significantly shorter in the quadruple mutant than WT. These results suggested that the dwarfed plant height of the quadruple mutant was caused by a shorter length. We also analyzed the plant height of the quadruple mutant and WT from 20 to 35 DAE (Fig. 4e). The result showed that the height of the quadruple mutant of *GmLHY* was shorter from 20 to 35 DAE.

### The quadruple mutant of *GmLHY* is deficient in the GA biosynthesis pathway

**Fig. 5:**
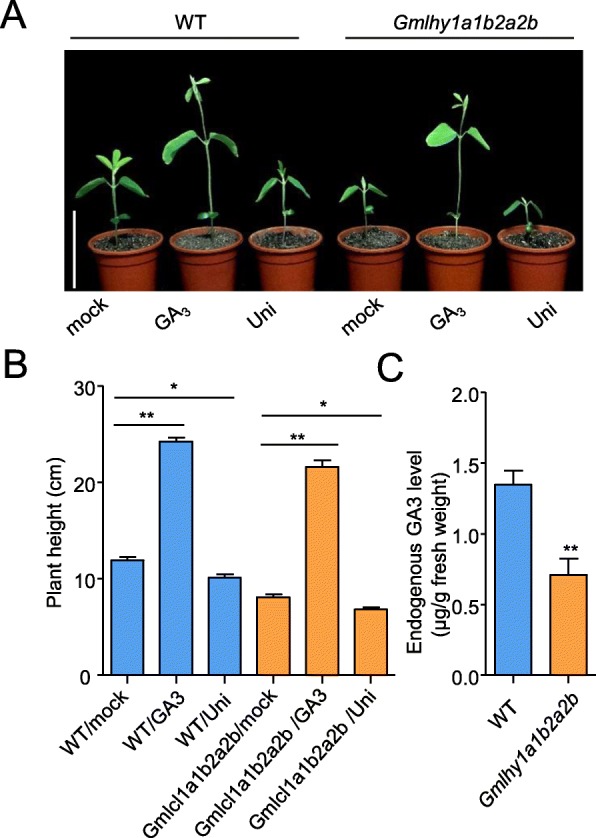
The quadruple mutant of *GmLHY* is a GA-deficient soybean mutant. **a** The morphological phenotypes of WT and quadruple mutant 4d past the exogenous GA3 (1 mg/L) and Uni (1 mg/L) application. **b** The statistical data of plant height of WT and quadruple mutant 4d past the exogenous GA3 (1 mg/L) and Uni (1 mg/L) application. The experiment was performed using three biological replicates, and differences were statistically analyzed using Student’s *t-*test (*P < 0.05; **P < 0.01). Bars indicate the s.e.m. **c** Determination of endogenous GA3 levels in the leaves of 20-day-old WT and quadruple mutant. The experiment was performed using three biological replicates, each with three technical replicates, and differences were statistically analyzed using Student’s *t*-test (**P < 0.01). Bars indicate the s.e.m

Previous studies showed that GAs is one of the most important phytohormones determining plant height [41, 42]. To test whether *GmLHY* affects the GA biosynthesis pathway, the *Gmlhy1a1b2a2b* mutant and WT were treated with GA_3_ and Uni (uniconazole, a GA biosynthesis inhibitor). The results showed that exogenous GA_3_ could restore the *Gmlhy1a1b2a2b* mutant to the WT, and Uni treatment could reduce the plant height of the WT and *Gmlhy1a1b2a2b* mutant seedlings (Fig. 5a, b). Endogenous GA_3_ levels from both the WT and *Gmlhy1a1b2a2b* mutant were determined using liquid chromatography–mass spectrometry (LC-MS). The results suggested that the levels of endogenous *GA3* in *Gmlhy1a1b2a2b* were lower than in WT (Fig. 5c). These findings indicated that the *Gmlhy1a1b2a2b* mutant has a low active gibberellin level and that it is a GA biosynthesis-deficient mutant.

### Expression analysis of GA metabolic pathway-related genes in the quadruple mutant of *GmLHY* and WT plants

**Fig. 6:**
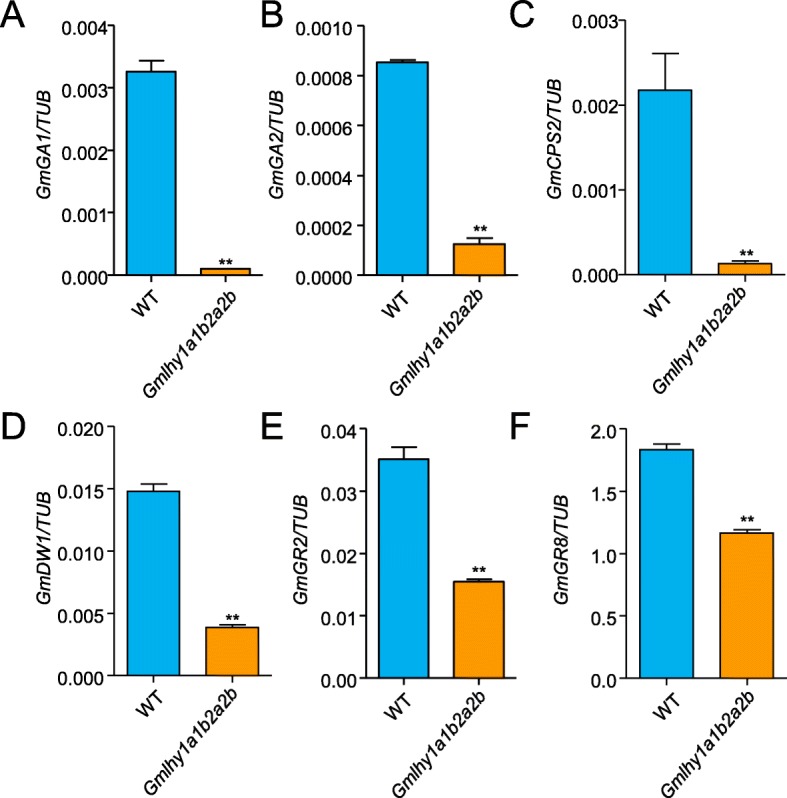
The relative expression of GA metabolic pathway-related genes in the quadruple mutant of *GmHY* and WT soybean plants. **a**–**d**. The expression level of GA biosynthesisrelated genes; **e**–**f** The expression level of GA response-related genes. Soybean *GmTUB* was used as an internal control to normalize all data. The experiment was performed using three biological replicates, and differences were statistically analyzed using Student’s *t-*test (**P < 0.01). Bars indicate the s.e.m.

Next, qRT-PCR was performed to measure the relative expression of genes that are known to participate in GA biosynthesis, such as GA-20 oxidase (*GmGA1*, *Glyma.09G149200*; *GmGA2*, *Glyma.20G153400*), copalyl pyrophosphate synthase (*GmCPS2*, *Glyma.19G157000*), ent-kaurene synthase (*GmDW1*, *Glyma.08G163900*), and GA-responsive genes (*GmGR2*, *Glyma.20G230600*; *GmGR8*, *Glyma.11G216500*) [13] in WT and the quadruple mutant of *GmLHY*. Compared with the WT plants, these genes showed significantly decreased expression in the quadruple mutant of *GmLHY* (Fig. 6a–f). Our findings suggested that *GmLHY* might positively regulate the expression of these GA biosynthesis and GA responsive genes, thereby limiting soybean plant height.

### Development of genetic markers and inheritance of quadruple mutant alleles

**Fig. 7:**
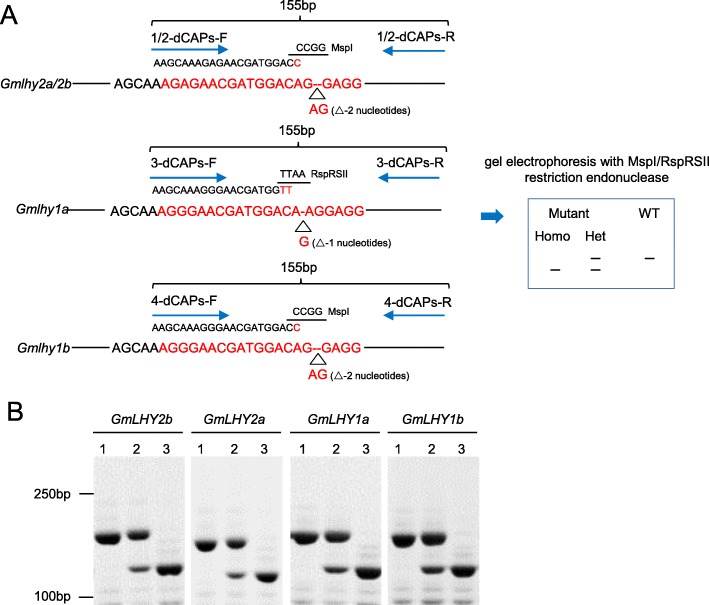
Inheritance and segregation of *GmLHY* gene small deletions. **a** An example of the dCAPs-specific primer designed for quadruple mutant of *GmLHY* is shown; **b** The genotyping of the quadruple mutant of *GmLHY* is shown. “1” indicates no gene change. “2” indicates that the gene was heterozygous. “3” indicates that the gene was homozygous

Genetic markers provide a critical and effective means of identifying mutant alleles for molecular-assisted studies and could possibly accelerate the genotyping procedure in future generations [38]. Therefore, we developed three dCAPs (Derived Cleaved Amplified Polymorphic Sequences) markers to identify the *Gmlhy1a1b2a2b* mutant alleles (Fig. 7a). For the genotyping of the *Gmlhy1a1b2a2b* mutants, PCR amplifications were performed using *GmLHY*-specific and dCAPs-specific primer pairs. The amplified products of *GmLHY2b*, *GmLHY2a,* and *GmLHY1b* on the mutant genomic DNA templates, but not on the WT genomic DNA templates, could be cleaved by the restriction endonuclease MspI (Fig. 7b). Additionally, the amplified products of *GmLHY1a* on the mutant genomic DNA templates, but not on the WT genomic DNA templates, could be cleaved by restriction endonuclease RspRSII (Fig. 7b). These results confirmed that the three dCAPs markers of *GmLHY* could be used for the genotyping of *Gmlhy1a1b2a2b* mutants and have further prospect in molecular breeding studies.

## Discussion

The CRISPR/Cas9 system is a recent development that has been rapidly and widely used to edit the genomes of various crops, such as soybean [37–39]. For example, Bao et al. obtained *GmSPL9* gene mutants using CRISPR/Cas9 and stable soybean transformation and found that the mutant of *GmSPL9s* demonstrated increased node number on the main stem and branch number, resulting in increased total node number per plants [38]. The CRISPR-edited soybean plants of both the *GmFAD2-1A* and *GmFAD2-1B* genes showed dramatic increases of over 80% in oleic acid content, whereas linoleic acid content decreased to 1.3–1.7% [39]. *LHY* and *CCA1* are important circadian clock genes that encode two morning-expressed MYB TFs in *Arabidopsis* [19, 20]. However, the functions of LHY/CCA1 family members in soybean are still unknown. In this study, we designed four target adaptors (target 1, target 2, target 3, and target 4) to edit four *GmLHY* genes (Fig. 1 a–c). In order to test whether the targets could perform properly in transgenic soybean plants, we first tested the CRISPR construct in transgenic soybean hairy roots using *Agrobacterium rhizogenes* strain K599. We confirmed that target 1 and target 3 could perform, while target 2 and target 4 might not work properly (Additional file 1: Fig. S2). We then performed stable soybean transformation and obtained 19 T_0_ events. In previous CRISPR/Cas9 research, chimeric mutations reduced the heritable transmission of mutant alleles in soybean [43, 44]. Therefore, in this study, we sought homozygous quadruple mutants of the *GmLHY* lines without transgenes and screened T_1_ plants derived from the T_0_ transgenic lines. Fortunately, we obtained one (T_1_–15) transgene-free homozygous quadruple mutant of *GmLHY* (Fig. 2Fc–f; Additional file 3: Table S2). Our findings demonstrated that the CRISPR/Cas9 system offers great potential in soybean breeding.

The circadian clock plays a critical role in the timing of multiple biological processes and stress responses in some model crops [16–18]. As key components of the circadian clock, LHY/CCA1 TFs have ability to initiate and set the phase of clock-controlled rhythms to produce a certain phenotype [16, 23, 24, 45, 46]. For example, the overexpression of *NaLHY* resulted in elongated hypocotyls and late flowering compared with WT plants in *Nicotiana attenuata* [23]. The same phenotypes were observed in *Arabidopsis AtLHY*-overexpressing lines [45, 46]. Although the functions of LHY/CCA1 were shown to be involved in flowering and stress responses in model crops, little is known regarding the biological functions of LHY/CCA1 family members in soybean. To explore the molecular function of genes in soybean, we examined the phenotype of the loss-of-function of *GmLHY* in the T_2_ transgene-free mutant. We found that the plant height in the *Gmlhy1a1b2a2b* mutant was shortened in soybean at 20 to 35 DAE (Fig. 4A–E). Our data demonstrated that the clock gene *GmLHY4*, as an MYB TF, functions in regulating plant height in soybean.

Plant height is generally considered to be a central yield trait for breeding in various crops [3–5]. GAs is a large group of tetracyclic diterpenoid plant hormones that regulate diverse biological processes in plant growth and development, such as embryogenesis, leaf primordia, flowering, and plant height [47–49]. In recent years, a few GA metabolic pathway-related genes associated with plant height have been reported in plants [13, 14]. For example, *SD1* encodes a gibberellin 20-oxidase gene (GA20oxs), and the reduced endogenous GA levels in the *sd1* mutant led to the short stature of rice variety IR8 [49, 50]. However, research on the molecular mechanisms of plant height regulation by TFs in soybean is lacking. In this study, the levels of endogenous GA3 in *Gmlhy1a1b2a2b* were lower than in WT, and the shortened internode phenotype could be rescued by treatment with exogenous GA3 (Fig. 5a-c). In addition, we tested the expression levels of GA synthetic genes (*GmDW1*, *GmGA1*, *GmGA2*, and *GmCPS2*) and GA response-related genes (*GmGR2* and *GmGR8*) in the quadruple mutant of *GmLHY* and WT soybean plants (Fig. 6a–f). We found that these genes had substantially decreased expression in the quadruple mutant of *GmLHY*. Overall, we speculated that GmLHY might positively regulate the expression of these GA metabolic pathway-related genes to reduce soybean plant height.

## Conclusions

The CRISPR/Cas9 system can be used for multiplex gene editing to advance crop plant breeding. In the present study, we used CRISPR/Cas9-based multiple genome editing to successfully obtain a quadruple mutant of *GmLHY* in soybean. Further, our results suggested that GmLHY directly or indirectly improves the expression level of GA synthetic genes and GA response-related genes to regulate soybean plant height. Our findings offer a case study for the use of gene editing to generate non-transgenic soybean genotypes and provide insight into the mechanisms underlying plant height regulatory networks in crop plant species.

## Methods

### Plasmid construction

The nucleotide sequences of the four *GmLHY* genes were downloaded from Phytozome (https://phytozome.jgi.doe.gov/pz/portal.html). The target sequences of the *GmLHY* genes were designed using the web tool CRISPR-P (http://cbi.hzau.edu.cn/crispr/). The pYLCRISPR/ Cas9P35S-B vector was a gift from Ma et al. [51]. The target sequences were subcloned into the different single guide RNA (sgRNA) expression cassettes and built into the pYLCRISPR/ Cas9P35S-B vector according to the protocol reported by Ma et al. [51]. The positive plasmids were introduced into *Agrobacterium tumefaciens* strain EHA101 for soybean stable transformation and into *Agrobacterium rhizogenes* strain K599 for soybean hairy roots transformation.

### Stable soybean transformation

The transformation procedure was according to a previous protocol [52, 53]. Putative transgenic soybean plants were screened by herbicide leaf-painting of T_0_ generation leaves at three vegetative stages (V3, V4, and V5) by wiping 100 mg/L^− 1^ glufosinate-ammonium solution onto the upper leaf surface. Genomic DNA was extracted from the leaves of herbicide-resistant plants using a NuClean Plant Genomic DNA Kit (CWBIO, China). To confirm the presence of the *Cas9* gene, PCR analysis was performed using *Cas9* gene-specific primers (Additional file 4: Table S3). The PCR amplifications were performed once for each DNA sample.

### *Agrobacterium rhizogenes*-mediated transformation of soybean hairy roots

Transgenic soybean hairy roots were generated by *A. rhizogenes*-mediated transformation as described by Kereszt et al. [40] and Cheng et al. [54] with some modifications. The cotyledons were cut into rough triangles and immediately placed into Petri dishes containing 0.8% agar medium to keep them moist. The cut surface was treated with 20 μL *A. rhizogenes* suspension. The dishes were sealed with Parafilm and placed in an incubator at 25 °C. Transformed hairy roots were abundant along a callus ridge on the inoculated cotyledons after approximately 2 weeks. The transgenic hairy roots were tested via PCR sequencing analysis.

### Identification of induced mutations using PCR and sequencing analyses

DNA was isolated from the transgenic soybean hairy roots and transgenic plants using a NuClean Plant Genomic DNA Kit (CWBIO, China). The regions spanning the targets of the *GmLHY* genes were amplified using KOD DNA Polymerase (Toyobo, Japan) with the different primer pairs in Additional file 4: Table S3. The sequences of the T_0_ and T_1_ generation plants and soybean hairy roots were analyzed using BioEdit to characterize the mutations induced by CRISPR/Cas9.

### Plant material, growth conditions, and primers

The soybean cultivar ‘Harosoy’ was used for soybean hairy root and stable transformations. To investigate the plant height of the transgenic plants, the T_2_ transgene-free mutants and WT control plants were grown in a growth chamber maintained at 25 °C and 70% relative humidity with a 16 h light/8 h dark cycle for 20–35 DAE. The node number on the main stem and internodal length were recorded at 20 DAE. The expression of GA biosynthesis genes and GA response-related genes was detected in the mutant and WT leaves at 20 DAE. All primers used for vector construction, PCR, and qRT-PCR assays for all target genes are listed in Additional file 4: Table S3.

### qRT-PCR analysis

Total RNA was isolated from the WT and T_2_ mutant soybean leaves using TRIzol reagent (Invitrogen, Shanghai, China). The cDNA synthesis was conducted using an M-MLV reverse transcriptase kit (Takara, Dalian, China) according to the manufacturer’s instructions. The qRT-PCR analysis was used to measure the transcript levels of the *GmLHY* genes, namely *GmGA1*, *GmGA2*, *GmCPS2*, *GmGR2*, *GmGR8*, and *GmDW1,* on a Roche LightCycler480 system (Roche, Germany) using a real-time PCR kit (Roche, Germany). The soybean housekeeping gene *GmTubllin* (*Glyma.05G157300*) was used as an internal reference to normalize all data. The relative transcript level of the target gene was calculated using the 2^−ΔΔCT^ method. Three biological replications per line were performed in each test.

### Molecular marker development

*GmLHY* sequences of the Harosoy and mutant genome were obtained by sequencing. Primers were designed using Primer Premier 5.0, with a product size < 200 bp. Three dCAPs markers were developed on the basis of the variations in the target 1/3 site of the *GmLHY* genes. *GmLHY2a* and *GmLHY2b* shared a pair of markers, and *GmLHY1a* and *GmLHY1b* each shared a pair of markers. Additional file 4: Table S3 lists the dCAPs markers that were used in this study.

### GA_3_ and Uni treatment, and endogenous GA determination

The *Gmlhy1a1b2a2b* mutant and WT were grown in a growth chamber at 25 °C under LD (16 h light/8 h dark) conditions, and 75% humidity. At approximately 20 DAE, 1 g (fresh weight) leaves tissue from the mutant or WT seedlings was harvested, weighed, immediately frozen in liquid nitrogen, and then stored at − 80 °C. The quantitative profiling of GA_3_ was determined using LC-MS. These analyses were conducted by the Suzhou Comin Biotechnology (Suzhou, China).

To assess the response of the *Gmlhy1a1b2a2b* mutant to GA_3_, 1.0 mg/L of GA_3_ was applied two times to seedlings with fully-open true leaves. The Uni (1.0 mg/L) treatment was carried out at the same time. The soybean growth condition was set as mentioned above. Three repeats were prepared for each treatment, and the effect of the hormone on stem expansion was evaluated 4 d later by measuring seedling length.

## Supplementary information

**Additional file 1: Figure S1.** Phylogenetic tree of LHY and CCA1 from *Arabidopsis* and soybean. The phylogenetic tree was inferred using the neighbor-joining method. The bootstrap consensus tree generated from 1000 replicates was used to represent the history of the different LHY/CCA1 proteins analyzed. **Figure S2.** CRISPR/Cas9-induced mutations of the four *GmLHY* genes in transgenic soybean hairy roots. A. Growth of transgenic hairy roots in the culture medium for 14 d. The typical lines were selected. B. Gel electrophoresis of PCR amplicons using specific primers for the CRISPR/Cas9 vector. C. Detailed sequence of the targets site in the transgenic soybean hairy roots. The red frames indicate the location of the targets. **Figure S3.** Sequencing of the CRISPR/Cas9-edited sites of *GmLHY* in the T_0_–7 line. A. Gel electrophoresis of the PCR amplicons using specific primers for CRISPR/Cas9 vector. B–E. The fragments containing the edited sites were amplified by PCR and directly sequenced. The sequencing chromatograms with superimposed peaks derived from biallelic mutations of the targeted sites were decoded by the DSD ecode program [51]. The red frames indicate the location of the targets.

**Additional file 2: Table S1.** CRISPR/Cas9-meditated targeted mutagenesis of four *GmLHY* genes in transgenic soybean hairy roots.

**Additional file 3: Table S2.** CRISPR/Cas9-meditated targeted mutagenesis of four *GmLHY* genes in transgenic soybean plants.

**Additional file 4: Table S3.** Primers used for PCR and qRT-PCR in this study.

## Data Availability

The datasets and materials developed and analyzed in this study are available from the corresponding author on reasonable request.

## Abbreviations

ABA: Abscisic acid
Br2: Brachytic2
Cas9: CRISPR-associated system 9
CPS2: Copalyl pyrophosphate synthase
CRISPR: Clustered regularly interspaced short palindromic repeat
DAE: Days after emergence
dCAPs: Derived cleaved amplified polymorphic sequences
DW1: Dwarf mutant
EE: Evening element
GA: Gibberellic acid
GA1/2: GA-20 oxidase1/2
GA3ox2: GA3 b-hydroxylase
GR2/8: GA-responsive gene 2/8
LC-MS: Liquid chromatography–mass spectrometry
LD: Long-day
LHY: LATE ELONGATED HYPOCOTYL
qRT-PCR: quantitative real-time PCR
TF: Transcription factor
TOC1: TIMING OF CAB EXPRESSION 1
WT: Wild type
