# Supplementary material for: CRISPR/Cas9-mediated targeted mutagenesis of GmLHY genes alters plant height and internode length in soybean
Source: BMC Plant Biol. 2019 Dec 18;19:562. doi: 10.1186/s12870-019-2145-8 (PMC6921449; doi:10.1186/s12870-019-2145-8)
Supplement: Supplementary file 1 — Additional file 1: Figure S1. Phylogenetic tree of LHY and CCA1 from Arabidopsis and soybean. The phylogenetic tree was inferred using the neighbor-joining method. The bootstrap consensus tree generated from 1000 replicates was used to represent the history of the different LHY/CCA1 proteins analyzed. Figure S2. CRISPR/Cas9-induced mutations of the four GmLHY genes in transgenic soybean hairy roots. A. Growth of transgenic hairy roots in the culture medium for 14 d. The typical lines were selected. B. Gel electrophoresis of PCR amplicons using specific primers for the CRISPR/Cas9 vector. C. Detailed sequence of the targets site in the transgenic soybean hairy roots. The red frames indicate the location of the targets. Figure S3. Sequencing of the CRISPR/Cas9-edited sites of GmLHY in the T0–7 line. A. Gel electrophoresis of the PCR amplicons using specific primers for CRISPR/Cas9 vector. B–E. The fragments containing the edited sites were amplified by PCR and directly sequenced. The sequencing chromatograms with superimposed peaks derived from biallelic mutations of the targeted sites were decoded by the DSD ecode program [51]. The red frames indicate the location of the targets. [file 12870_2019_2145_MOESM1_ESM.pdf]

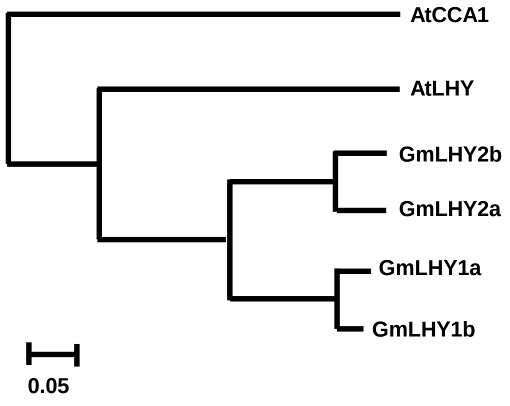

Figure S1. Phylogenetic tree of LHY and CCA1 from *Arabidopsis* and soybean. The phylogenetic tree was inferred using the neighbor-joining method. The bootstrap consensus tree generated from 1000 replicates was used to represent the history of the different LHY/CCA1 proteins analyzed.

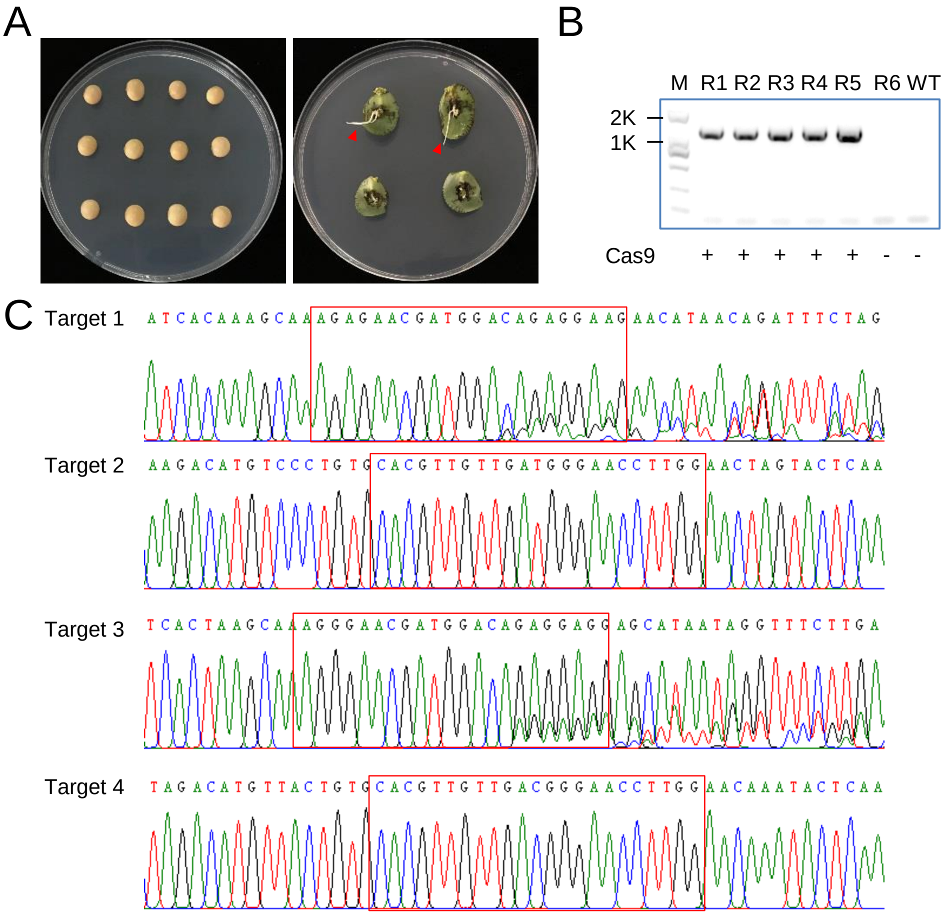

Figure S2. CRISPR/Cas9-induced mutations of the four *GmLHY* genes in transgenic soybean hairy roots. **A.** Growth of transgenic hairy roots in the culture medium for 14 d. The typical lines were selected. **B.** Gel electrophoresis of PCR amplicons using specific primers for the CRISPR/Cas9 vector. **C.** Detailed sequence of the targets site in the transgenic soybean hairy roots. The red frames indicate the location of the targets.

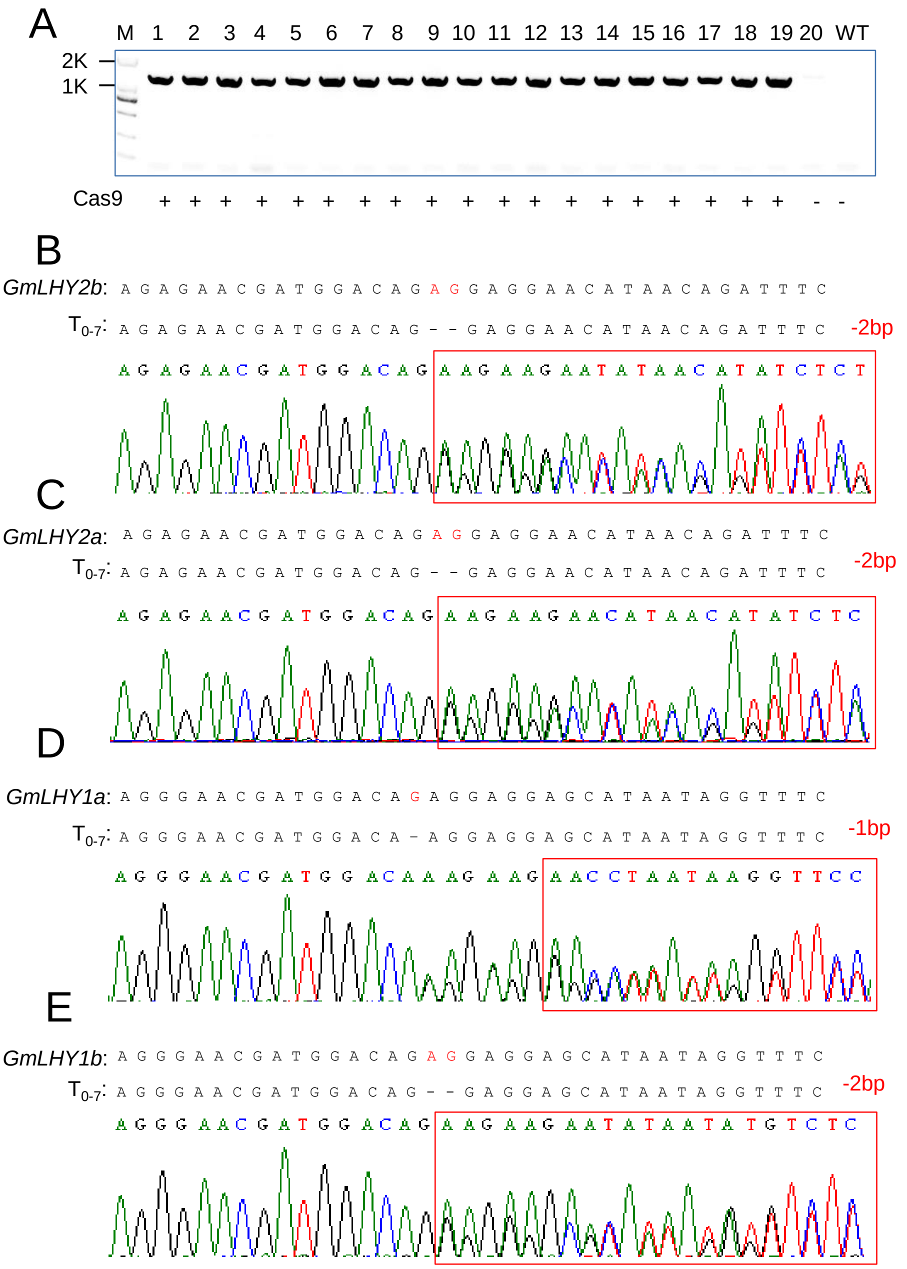

Figure S3. Sequencing of the CRISPR/Cas9-edited sites of *GmLHY* in the T_0-7 line. **A.** Gel electrophoresis of the PCR amplicons using specific primers for CRISPR/Cas9 vector. **B–E.** The fragments containing the edited sites were amplified by PCR and directly sequenced. The sequencing chromatograms with superimposed peaks derived from biallelic mutations of the targeted sites were decoded by the DSD ecode program [51]. The red frames indicate the location of the targets.
