# Supplementary material for: CRISPR/Cas9-mediated targeted mutagenesis of GmLHY genes alters plant height and internode length in soybean
Source: BMC Plant Biol. 2019 Dec 18;19:562. doi: 10.1186/s12870-019-2145-8 (PMC6921449; doi:10.1186/s12870-019-2145-8)
Supplement: Supplementary file 2 — Additional file 2: Table S1. CRISPR/Cas9-meditated targeted mutagenesis of four GmLHY genes in transgenic soybean hairy roots. [file 12870_2019_2145_MOESM2_ESM.doc]

| Plant ID | Target 1 | Target 2 | Target 3 | Target 4 | Cas9 |
| --- | --- | --- | --- | --- | --- |
| R1 | √ | × | √ | × | + |
| R2 | × | × | √ | × | + |
| R3 | √ | × | × | × | + |
| R4 | √ | × | √ | × | + |
| R5 | × | × | √ | × | + |
| R6 | × | × | × | × | - |

Table S1. CRISPR/Cas9-meditated targeted mutagenesis of four *GmLHY* genes in transgenic soybean hairy roots.

“×” represent gene was no changed. “√” represent gene was edited. “+” represent *Cas9* gene was positive. “-” represent *Cas9* gene was negative.
