# Supplementary material for: CRISPR/Cas9-mediated targeted mutagenesis of GmLHY genes alters plant height and internode length in soybean
Source: BMC Plant Biol. 2019 Dec 18;19:562. doi: 10.1186/s12870-019-2145-8 (PMC6921449; doi:10.1186/s12870-019-2145-8)
Supplement: Supplementary file 3 — Additional file 3: Table S2. CRISPR/Cas9-meditated targeted mutagenesis of four GmLHY genes in transgenic soybean plants. [file 12870_2019_2145_MOESM3_ESM.doc]

Table S2. CRISPR/Cas9-meditated targeted mutagenesis of four *GmLHY* genes in transgenic soybean plants.

| Plant ID | *GmLCL1* | *GmLCL2* | *GmLCL3* | *GmLCL4* | *Cas9* |
| --- | --- | --- | --- | --- | --- |
| T_0-1_  T_0-2_  T_0-3_  T_0-4_  T_0-5_  T_0-6_  T_0-7_  T_0-8_  T_0-9_  T_0-10_  T_0-11_  T_0-12_  T_0-13_  T_0-14_  T_0-15_  T_0-16_  T_0-17_  T_0-18_  T_0-19_  T_0-20_  T_1-1_  T_1-2_  T_1-3_  T_1-4_  T_1-5_  T_1-6_  T_1-7_  T_1-8_  T_1-9_  T_1-10_  T_1-11_  T_1-12_  T_1-13_  T_1-14_  T_1-15_  T_1-16_  T_1-17_  T_1-18_  T_1-19_  T_1-20_  T_1-21_  T_1-22_ | H  ×  ×  ×  ×  H  H  ×  ×  H  ×  ×  H  H  H  ×  ×  ×  ×  ×  H  ×  H  ×  H  ×  √  H  ×  √  √  H  H  H  √  H  ×  √  ×  ×  H  × | ×  H  ×  ×  H  ×  H  ×  H  ×  ×  ×  ×  H  ×  H  ×  ×  ×  ×  H  ×  √  H  H  H  √  H  ×  H  H  H  ×  H  √  ×  ×  √  ×  √  ×  × | H  H  ×  H  ×  ×  H  ×  ×  ×  H  H×  ×  ×  ×  H  ×  ×  H  ×  ×  ×  ×  √  H  ×  √  H  ×  H  H  H  ×  H  √  ×  H  ×  ×  H  ×  × | ×  ×  ×  H  ×  ×  H  ×  ×  ×  H  ×  ×  ×  ×  ×  ×  ×  ×  ×  ×  ×  √  √  H  H  √  ×  ×  H  H  H  H  H  √  ×  H  H  H  H  ×  × | +  +  +  +  +  +  +  +  +  +  +  +  +  +  +  +  +  +  +  +  +  -  +  -  +  -  +  +  -  +  -  +  +  +  -  +  +  +  +  -  +  - |

“H” represent gene was heterozygous. “×” represent gene was no changed. “√” represent gene was homozygous. “+” represent *Cas9* gene was positive. “-” represent *Cas9* gene was negative.
