# Supplementary material for: CRISPR/Cas9-mediated targeted mutagenesis of GmLHY genes alters plant height and internode length in soybean
Source: BMC Plant Biol. 2019 Dec 18;19:562. doi: 10.1186/s12870-019-2145-8 (PMC6921449; doi:10.1186/s12870-019-2145-8)
Supplement: Supplementary file 4 — Additional file 4: Table S3. Primers used for PCR and qRT-PCR in this study. [file 12870_2019_2145_MOESM4_ESM.doc]

Table S3 Primers used for PCR and qRT-PCR in this study.

Primers for constructs and sequencing

| Primers name | Primers sequences (5’-3’) |
| --- | --- |
| Target 1- F | AGAGAACGATGGACAGAGGAGG |
| Target 1- R | CCTCCTCTGTCCATCGTTCTCT |
| Target 2- F | CACGTTGTTGATGGGAACCTAGG |
| Target 2- R | CCTAGGTTCCCATCAACAACGTG |
| Target 3- F | AGGGAACGATGGACAGAGGAGG |
| Target 3- R | CCTCCTCTGTCCATCGTTCCCT |
| Target 4- F | CACGTTGTTGACGGGAACCTAGG |
| Target 4- R | CCTAGGTTCCCGTCAACAACGTG |
| GmLHY2b- F | GCTTTACTATTTGGTGTGTTCCATG |
| GmLHY2b- R | TTGACATGGCATGGGACAAGAGT |
| GmLHY2a- F | GGTCCGAAGGAAAGTCTCACG |
| GmLHY2a- R | TCTGAGCATGACTCCTGATTTGC |
| GmLHY1a- F | GAGAAAATTGAAGCGAACTGCG |
| GmLHY1a- R | CCCATTGTTGCTAGAACCCC |
| GmLHY1b- F | GTTTCTTTTGCAGTAACATCATCAC |
| GmLHY1b- R | TGGGTTCTGATACTGAAGAAGCG |
| Cas9- F | GTGGGAGGCGAGGTAGAG |
| Cas9- R | CGTCGACCACATTGTTCCTC |

Primers for quantitative real-time PCR

| Primers name | Primers sequences (5’-3’) |
| --- | --- |
| qGmLHY2b- F | AGAACGAGGAGAGATTAAACCACA |
| qGmLHY2b- R | GGGTTCTCAGAAATTGCTTGGT |
| qGmLHY2a- F | TGGATCCAGAACACTCAGAAGC |
| qGmLHY2a- R | TCGGGGTTCTCAGAAATGACC |
| qGmLHY1a- F | GGCCTTTCAGGCTCTGTTCT |
| qGmLHY1a- R | AACTGCATTTCTTGCTGCCG |
| qGmLHY1b- F | CTGTTCTCCTGCATCGGTGA |
| qGmLHY1b- R | ATGGCAGCTGGTGACTTTGA |
| GmTubllin- F | TCTTGGACAACGAAGCCATCT |
| GmTubllin- R | GGTGAGGGACGAAATGATCT |
| GmGA1-F | GATAGAGAGACCCTGTGCCT |
| GmGA1-R | TGAGAAGCAGAGCAAAACAGAG |
| GmGA2-F | TGG CTGCAACGGAAAAGTAA |
| GmGA2-R | TAGCCCCATAGCCCTACTCA |
| GmGR2-F | AGTTCCTGTATCCCTGTGCC |
| GmGR2-R | TGGCAGGGAAAGAGAAGAGG |
| GmGR8-F | TCCCCAGATCGTTACCATCG |
| GmGR8-R | TCCCAAGGTACAACTCGGAC |
| GmCPS2-F | ACTGCCACCTTCCCTCTTTC |
| GmCPS2-R | TGTTTGTCGTTAGTCTCGGAC |
| GmDW1-F | ATGTGCTGGCTTTGCGTATT |
| GmDW1-R | CCTTGCACTCTCTGGGAACT |

Primers for genetic markers

| Primers name | Primers sequences (5’-3’) |
| --- | --- |
| 1/2- dCAPs-F | AAGCAAAGAGAACGATGGACC |
| 1/2- dCAPs-R | TAAAATGCATTTATGCATCTGAA |
| 3- dCAPs-F | AAGCAAAGGGAACGATGGTT |
| 3- dCAPs-R | TCCAGCTTTGTAAAGAACTTCT |
| 4- dCAPs-F | AAGCAAAGGGAACGATGGACC |
| 4- dCAPs-R | TACACCAATGTTTAGAAAGG |
